# A Model of Aerobic and Anaerobic Metabolism of Hydrogen in the Extremophile *Acidithiobacillus ferrooxidans*

**DOI:** 10.3389/fmicb.2020.610836

**Published:** 2020-11-30

**Authors:** Jiri Kucera, Jan Lochman, Pavel Bouchal, Eva Pakostova, Kamil Mikulasek, Sabrina Hedrich, Oldrich Janiczek, Martin Mandl, D. Barrie Johnson

**Affiliations:** ^1^Department of Biochemistry, Faculty of Science, Masaryk University, Brno, Czechia; ^2^School of Biological Sciences, College of Natural Sciences, Bangor University, Bangor, United Kingdom; ^3^Mendel Centre for Plant Genomics and Proteomics, Central European Institute of Technology, Brno, Czechia; ^4^Institute of Biosciences, Technische Universität (TU) Bergakademie Freiberg, Freiberg, Germany

**Keywords:** *Acidithiobacillus*, extremophiles, ferric iron reduction, hydrogen metabolism, multi-omics, oxygen reduction

## Abstract

Hydrogen can serve as an electron donor for chemolithotrophic acidophiles, especially in the deep terrestrial subsurface and geothermal ecosystems. Nevertheless, the current knowledge of hydrogen utilization by mesophilic acidophiles is minimal. A multi-omics analysis was applied on *Acidithiobacillus ferrooxidans* growing on hydrogen, and a respiratory model was proposed. In the model, [NiFe] hydrogenases oxidize hydrogen to two protons and two electrons. The electrons are used to reduce membrane-soluble ubiquinone to ubiquinol. Genetically associated iron-sulfur proteins mediate electron relay from the hydrogenases to the ubiquinone pool. Under aerobic conditions, reduced ubiquinol transfers electrons to either cytochrome *aa*_3_ oxidase via cytochrome *bc*_1_ complex and cytochrome *c*_4_ or the alternate directly to cytochrome *bd* oxidase, resulting in proton efflux and reduction of oxygen. Under anaerobic conditions, reduced ubiquinol transfers electrons to outer membrane cytochrome *c* (ferrireductase) via cytochrome *bc*_1_ complex and a cascade of electron transporters (cytochrome *c*_4_, cytochrome *c*_552_, rusticyanin, and high potential iron-sulfur protein), resulting in proton efflux and reduction of ferric iron. The proton gradient generated by hydrogen oxidation maintains the membrane potential and allows the generation of ATP and NADH. These results further clarify the role of extremophiles in biogeochemical processes and their impact on the composition of the deep terrestrial subsurface.

## Introduction

Microbial life in the deep terrestrial subsurface is a subject of considerable interest, as geochemical processes provide a source of energy for the metabolism of chemolithotrophic microbial communities. While the deep continental subsurface is estimated to contain up to 20% of the Earth’s total biomass (McMahon and Parnell, 2014), this biosphere is one of the least understood ecosystems because of the methodological limitations of studying niches dispersed in solid matrixes (Amils, 2015). Understanding the nature of deep terrestrial biospheres and how these are maintained is fundamental to deciphering the origin of life not only on Earth, but potentially on other planets and moons (Chapelle et al., 2002; Bauermeister et al., 2014). In addition, microbial processes impact the geochemistry of deep repositories and groundwater reservoirs, affecting the feasibility of resource extraction. Since raw materials, such as metal ores, located close to Earth’s surface are becoming depleted, the exploration of deep-buried (>1 km) mineral deposits is currently focused on the deeper subsurface (Johnson, 2015). Subsurface life is dependent on buried organic matter and geogenic reduced compounds such as hydrogen gas (H_2_) (Stevens, 1997; Bagnoud et al., 2016). Hydrogen in the subsurface can be generated by the process of hydration (serpentinization) of an igneous rock with a very low silica content and rich in minerals (ultramafic rock) (Mayhew et al., 2013) and the radiolysis of water (Blair et al., 2007). In addition, many anaerobic bacteria can exploit organic compounds to produce H_2_ by reducing protons (Teng et al., 2019). However, the importance of H_2_ as an electron donor for acidophiles in the subsurface and geothermal springs as well as deep-sea hydrothermal vents remains unknown. Also, most of the information that has been published on acidophilic life in the subterranean environments has come from the research of abandoned deep mines and caves (Johnson, 2012). Recently, drill cores taken from the largest known massive sulfide deposit have confirmed the presence of members of hydrogen, methane, iron and sulfur oxidizers, and sulfate-reducers many of which are acidophilic (Puente-Sánchez et al., 2014, 2018).

Four distinct bacterial phyla containing a number of acidophiles (*Actinobacteriota*, *Acidobacteriota*, *Chloroflexota*, and *Verrucomicrobiota*) have been experimentally shown to utilize atmospheric H_2_ by the [NiFe] group 2a hydrogenase (Islam et al., 2020). To date, autotrophic growth by dissimilatory H_2_ oxidation has been reported only for several acidophiles. Among the acidophilic archaea, members of the genera *Sulfolobus*, *Acidianus*, and *Metallosphaera* were found to be able to grow aerobically on H_2_ (Huber et al., 1992). Acidophilic bacteria growing aerobically on H_2_ include obligate autotrophs *Acidithiobacillus* spp. (iron/sulfur-oxidizing *At. ferrooxidans*, *At. ferridurans*, *At. ferrianus*, and sulfur-oxidizing *At. caldus*), iron/sulfur-oxidizing facultative autotrophs *Sulfobacillus* spp. (*Sb. acidophilus*, *Sb. benefaciens*, and *Sb. thermosulfidooxidans*), and iron-oxidizing facultative autotroph *Acidimicrobium ferrooxidans* (Drobner et al., 1990; Ohmura et al., 2002; Hedrich and Johnson, 2013; Norris et al., 2020). Of these, *At. ferrooxidans*, *At. ferridurans*, *At. ferrianus*, *Sb. thermosulfidooxidans*, and *Sb. benefaciens* have been reported to grow anaerobically using H_2_ as an electron donor and Fe^3+^ as an electron acceptor (Ohmura et al., 2002; Hedrich and Johnson, 2013; Norris et al., 2020). Although genomic studies have demonstrated the presence of genes encoding different hydrogenases in many acidophiles, their presence does not necessarily mean that these bacteria grow by oxidizing H_2_, as some hydrogenases may produce H_2_ (Valdés et al., 2008). Hydrogen metabolism can be divided into the respiratory oxidation of H_2_ to H^+^ (uptake) linked to quinone reduction in membrane-bound respiratory electron transfer chain, and H_2_ production by reducing H^+^ to H_2_ in non-energy conserving anaerobic system with a low electron transfer potential. The redox reactions are catalyzed by metalloenzymes (hydrogenases) (Lubitz et al., 2014). The transport of electrons to or from H_2_ is associated with H^+^ translocation across the membrane, which results in energy conservation in the form of proton motive force (PMF). Hydrogenases consist of three phylogenetically distinct classes, i.e., [NiFe], [FeFe], and [Fe] hydrogenases (Vignais and Billoud, 2007). The reduction potentials of the active site and prosthetic groups of [NiFe] hydrogenase from *Allochromatium vinosum* were determined to range from –390 to –30 mV (Armstrong and Albracht, 2005). The *At. ferrooxidans* ATCC 23270^T^ genome has been shown to encode four different types of [NiFe] hydrogenases: (i) membrane-bound respiratory [NiFe] group 1 hydrogenase, (ii) cyanobacterial uptake and cytoplasmic [NiFe] group 2 hydrogenase, (iii) bidirectional hetero-multimeric cytoplasmic [NiFe] group 3 hydrogenase, (iv) H_2_-evolving, energy-conserving, membrane-associated [NiFe] group 4 hydrogenase (Valdés et al., 2008). Even though H_2_ as an electron donor has several advantages for acidophiles compared to other inorganic substrates, including avoiding generating or consuming acidity and Fe^3+^ precipitation, few physiological studies have been reported (Fischer et al., 1996; Ohmura et al., 2002; Islam et al., 2020), and no detailed information on H_2_ uptake and metabolic pathways in acidophilic mesophiles is available. Recently, the energy metabolism pathways for autotrophic growth on H_2_ in thermoacidophilic methanotrophs of the genus *Methylacidiphilum* and *Methylacidimicrobium* (both *Verrucomicrobia*) from extremely acidic geothermal systems have been proposed (Carere et al., 2017; Mohammadi et al., 2019; Schmitz et al., 2020).

*At. ferrooxidans* contains three types of membrane-bound terminal oxidases, including an *aa*_3_ cytochrome *c* oxidase and cytochrome ubiquinol oxidases of the *bd* and *bo*_3_ type. The level of their expression was found to be dependent on whether ferrous iron or zero-valent sulfur was provided as electron donor (Quatrini et al., 2009). The *aa*_3_ cytochrome *c* oxidase that is part of the *rus* operon was shown to be induced when the *At. ferrooxidans* cells oxidized Fe^2+^ aerobically, while the *bd* and *bo*_3_ cytochrome ubiquinol oxidases were induced during aerobic S^0^ oxidation. Although optical spectra of *At. ferrooxidans* cells grown with S^0^ showed higher intensity of peaks with an absorption maximum at 613 nm, indicating the *ba*_3_ type cytochrome *c* oxidase (Brasseur et al., 2004), the corresponding genes which encode this complex have not been found in the *At. ferrooxidans* genomes sequenced to date. The mechanism of respiratory Fe^3+^ reduction in *Acidithiobacillus* spp. has not been fully elucidated. It has been assumed that Fe^3+^ reduction occurs outside of the inner membrane due to the insolubility of Fe^3+^ above pH ∼2.5, and the toxicity of elevated concentrations of ferrous iron (Corbett and Ingledew, 1987). However, no respiratory Fe^3+^ reductase has been confirmed biochemically in *Acidithiobacillus* spp. to date. Tetrathionate hydrolase and arsenical resistance protein, both of which were previously suggested to mediate Fe^3+^ reduction in *At. ferrooxidans* (Sugio et al., 2009; Mo et al., 2011), were not detected during anaerobic oxidation of S^0^ coupled to Fe^3+^ reduction (Kucera et al., 2012, 2016b; Osorio et al., 2013), and also in this study. It follows, therefore, that respiratory Fe^3+^ reduction is mediated by another enzyme(s). An indirect mechanism has also been proposed involving the non-enzymatic reduction of Fe^3+^ by H_2_S generated by a disproportionation of S^0^ which is mediated by sulfur reductase during anaerobic growth with elemental sulfur (Osorio et al., 2013). However, this mechanism is not relevant during growth with H_2_. As early as the 1980s, the same cytochromes and electron transporters involved in the aerobic Fe^2+^ oxidation were suggested to mediate anaerobic Fe^3+^ reduction, but in reverse (Corbett and Ingledew, 1987). This hypothesis was subsequently confirmed by several transcriptomic and proteomic approaches in *At. ferrooxidans* grew anaerobically on S^0^ coupled to Fe^3+^ reduction (Kucera et al., 2012, 2016a,b; Osorio et al., 2013; Norris et al., 2018). The main proposed multiple mechanisms included products of the *rus* operon such as the outer membrane cytochrome *c* (Cyc2, expected to function as a terminal Fe^3+^ reductase), the periplasmic electron transporters rusticyanin and cytochrome *c*_552_ (Cyc1), as well as products of the *petI* and *petII* operons such as the periplasmic high potential iron-sulfur protein Hip, cytochromes *c*_4_ (CycA1 and CycA2), and the inner-membrane cytochrome *bc*_1_ complexes I and II (PetA1B1C1 and PetA2B2C2), with the UQ/UQH_2_ pool providing a connection to the electron donor oxidation (Kucera et al., 2016a). In addition, the loss of the ability to anaerobically reduce Fe^3+^ was observed in *At. ferrooxidans* subcultures subsequently passaged aerobically on elemental sulfur (Kucera et al., 2016b). Further analysis revealed dramatic changes within *rus*, *petI*, and *petII* operons products, resulting in a decrease in Cyc2 and Rus at both the RNA and protein levels, and down-regulation of *cyc1, cycA1, petA1*, and *cycA2* (Kucera et al., 2016b). The loss of the ability to anaerobically reduce Fe^3+^ was also observed in *At. ferridurans*, which was caused by salt stress-induced insertional inactivation of the *rus* operon (Bonnefoy et al., 2018). This evidence pointed to the essential role of some gene(s) encoded by the *rus* operon in the mechanism of anaerobic respiratory Fe^3+^ reduction in iron-oxidizing acidithiobacilli.

In this study, a multi-omics approach, involving transcriptomics and proteomics, was used to reveal the respiratory pathways in the mesophilic acidophile *At. ferrooxidans* oxidizing H_2_ as the sole electron donor under both aerobic (coupled to oxygen reduction) and anaerobic (coupled to ferric iron reduction) conditions.

## Materials and Methods

### Bacterial Strains and Growth Conditions

*At. ferrooxidans* strain CCM 4253 (GCA_003233765.1) was plated onto a selective overlay medium containing ferrous sulfate (Johnson and Hallberg, 2007) and incubated aerobically at 30°C for 10 days, after which a single colony was transferred into a sterile medium containing basal salts and trace elements (Osorio et al., 2013), adjusted with sulfuric acid to pH 1.9. The re-purified culture was grown both aerobically and anaerobically with H_2_ as sole electron donor in 1 L shake flasks containing 500 mL basal salts medium placed in 2.5 L sealed jars (Oxoid, United Kingdom), where the atmosphere was enriched with both H_2_ and CO_2_, as described elsewhere (Hedrich and Johnson, 2013). In brief, 1.3 g sodium bicarbonate, 0.3 g sodium borohydride and 0.15 g citric acid was put into 20 mL universal bottles, 10 mL of water added, and the effervescing mixture placed into the jars, which were sealed as rapidly as possible. This generated an atmosphere containing up to 31.7 mmoles of H_2_ and 15.5 mmoles of CO_2_ (some of the nascent gases were invariably lost during sealing of the jars). The sealed jars were maintained at 30°C and agitated. Initially *At. ferrooxidans* was adapted to aerobic growth on H_2_ (where the sealed jars contained 23.0 mmoles of O_2_), and subsequently to anaerobic conditions where Fe^3+^ (25 mmoles; added from a 1 M filter-sterilized stock solution of ferric sulfate, pH 1.5) replaced oxygen as terminal electron acceptor. For the latter, O_2_ was removed by placing an AnaeroGen^TM^ sachet (Oxoid) into each jar. This caused the O_2_ present to be reduced to CO_2_, producing an atmosphere containing up to 38.5 mmoles of CO_2_. Ferrous iron production was monitored using the ferrozine colorimetric method (Stookey, 1970). After almost all Fe^3+^ was reduced, the cultures were harvested by centrifugation at 15,000 × *g* for 20 min at 4°C. The resulting pellets were washed with sterile dilute sulfuric acid (pH 1.7), frozen and stored at −70°C until further processing. Control triplicate aerobic cultures were grown in shake flasks (500 ml in 1 L conical flasks) in the basal salts medium as described above, containing 0.05 mmoles Fe^2+^, and supplemented with 31.25 mmoles magnesium sulfate heptahydrate, to compensate the osmotic stress caused by including ferric sulfate in anaerobic cultures. When the biomass densities reached 10^9^ cells mL^–1^, the cultures were centrifuged at 15,000 × *g* for 20 min at 4°C, pellets frozen and stored at −70°C. Cells were enumerated using a Thoma counting chamber and a Leitz Wetzlar 766200 (Germany) phase-contrast microscope.

### RNA Sequencing and Transcript Analysis

Three biological replicates were prepared for the aerobic and anaerobic H_2_-grown *At. ferrooxidans*. The total RNA was extracted with a TRI reagent (Sigma-Aldrich) and was treated with a TURBO DNA-free kit (Ambion) to remove the contaminating DNA. The quality and quantity of RNA were assessed with a Qubit fluorometer (Thermo Fisher Scientific) and by Agilent 4200 TapeStation (Agilent). The cDNA libraries were constructed using the ScriptSeq^TM^ Complete Kits (Bacteria) (Illumina), including Ribo-Zero^TM^ technology for ribosomal RNA removing and ScriptSeq v2 RNA-Seq Library Preparation Kit. Sequencing was performed on the Illumina MiSeq platform with MiSeq Reagent Kit v2 (500 cycles), which generated 250 bp paired-end reads. Quality control of reads was performed using R package ShortRead (Morgan et al., 2009). Subsequently, reads were aligned to the genome sequence of the *At. ferrooxidans* CCM 4253 (GCA_003233765.1) using the software BBmap (Bushnell et al., 2017). R package DeSeq2 was used for differential analysis of count data (Love et al., 2014). Transcripts with |log_2_ fold change| > 1 and *q* < 0.05 (FDR-adjusted *P*-values) were considered as differentially expressed genes (DEGs).

### MS Proteomics and Protein Identification

Three biological replicates for each condition were used for proteomic analyses. 200 μl of lysis buffer containing 8 M urea and 0.1 M Tris-HCl (pH 7.5) was added to each bacterial pellet of aerobically and anaerobically grown cells. The suspensions were homogenized by needle sonication (90 × 0.5 s pulses at 50 W; HD 2200, Bandelin) and then incubated for 60 min at room temperature. Homogenates were centrifuged at 14,000 × *g* for 20 min at 4°C and the supernatants (protein lysates) were stored at −80°C. The protein concentration was determined by RC-DC Protein Assay (Bio-Rad). One hundred μg of protein lysates were digested with trypsin (Promega; 1:30 trypsin:protein ratio) on 30 kDa Microcon columns (Merck Millipore) as previously described (Janacova et al., 2020). The eluted peptides were desalted on a C18 column (MicroSpin, Harvard Apparatus) (Bouchal et al., 2009), dried and stored at −80°C. Prior to LC-MS analysis, the peptides were transferred into LC-MS vials using acidic extraction and concentrated in a vacuum concentrator to 25 μL (Hafidh et al., 2018). The peptide concentration was assessed using LC-UV analysis on the RSLCnano system (Thermo Fisher Scientific) based on the area under the UV chromatogram (214 nm) using an external calibration curve using in-house MEC cell line lysate digest (from 50 to 2,000 ng per injection). One to two microliters of concentrated sample were spiked in with 2 μL of 10-fold diluted iRT peptide mix (Biognosys) for data dependent acquisition (DDA), or data independent acquisition (DIA), respectively. The sample volume was adjusted to 10 μL total volume by the addition of 0.5% (v/v) formic acid and 0.001% (w/v) poly(ethylene glycol) (PEG 20,000) (Stejskal et al., 2013). Then, 5 μL of the 10-, or 5-fold diluted samples corresponding to approximately 0.5, or 1.0 μg of peptide material was injected onto a column for DDA, or DIA analyses, respectively. LC-MS/MS analyses of diluted peptide mixtures with spiked in iRT peptides were performed using an RSLCnano System coupled to a TOF Impact II mass spectrometer (Bruker Daltonics). Before LC separation, the samples were concentrated online on the trap column (100 μm × 20 mm) filled with 5 μm, 100 Å, C18 sorbent (Thermo Fisher Scientific, Waltham). The trapping and analytical columns were equilibrated before injecting the sample into the sample loop. The peptides were separated using an Acclaim Pepmap100 C18 column (3 μm particles, 100 Å, 75 μm × 500 mm; Thermo Fisher Scientific) at a flow rate of 300 nL min^–1^ with the following LC gradient program, where the mobile phase A was 0.1% (v/v) FA in water and mobile phase B was 0.1% (v/v) FA in 80% (v/v) acetonitrile: the proportion of mobile phase B was increased from the initial value of 1–56% over 120 min, raised to 90% between 120 and 130 min, and then held at 90% for 10 min. The analytical column’s outlet was directly connected to a CaptiveSpray nanoBooster ion source (Bruker Daltonics). Each sample was analyzed in DDA mode for spectral library generation, and DIA mode for DIA-based quantification. In DDA mode, the NanoBooster was filled with acetonitrile, and then MS and MS/MS spectra were acquired with a 3 s cycle time. The mass range was set to 150–2,200 m/z, and precursors were selected from 300 to 2,000 m/z. The acquisition speeds of the MS and MS/MS scans were 2 and 4–16 Hz, respectively, with the precise speed for MS/MS acquisitions being based on precursor intensity. For protein quantification in all samples in DIA mode, the NanoBooster was bypassed. MS and MS/MS data were acquired by performing survey MS scan followed by 64 MS/MS scans variable SWATH windows (Collins et al., 2017) between 400 and 1,200 m/z (1 m/z overlap). The acquisition speed of MS/MS scans was 20 Hz, the speed of MS/MS spectrum acquisition depended on precursor intensity, and the cycle time did not exceed 3.5 s. To create a spectral library, DDA data were searched in MaxQuant 1.5.8.3.^1^ against the genome sequence of the *At. ferrooxidans* CCM 4253 (GCA_003233765.1) complemented with the iRT protein database (Biognosys) and the internal database of common protein contaminants in Andromeda using the default settings for a Bruker qTOF-type mass spectrometer. In these searches, trypsin was the designated enzyme (cleaving polypeptides on the carboxyl side of lysine or arginine except when either is followed by proline), the maximum missed cleavage sites were set to 2, and the taxonomy was set as *At. ferrooxidans*. The PSM, protein, and site FDR thresholds were all set to 0.01 based on decoy database search. The precursor and fragment mass tolerances were set to 0.07 Da/0.006 Da (first search/main search) and 40 ppm, respectively. The permitted dynamic modifications were Oxidation (M); Acetyl (Protein N-terminus), and the only permitted static (fixed) modification was Carbamidomethyl (C). The spectral library was created in Spectronaut 11.0 (Biognosys), based on MaxQuant search results for all DDA analyses; it contained 14,331 precursors representing 11,409 peptides (of these, 11,051 were proteotypic), 1,620 protein groups and 1,658 proteins. The spectral library file is available in the PRIDE dataset. Quantitative information was extracted from the DIA data using Spectronaut 11.0 for all corresponding proteins/peptides/transitions and all conditions, using an algorithm implemented in Spectronaut. Only proteotypic peptides detected with significant confidence (*q* < 0.01) at least three times across all DIA runs were included in the final dataset; this was ensured by using the “*q*-value 0.5 percentile” setting in Spectronaut. Local data normalization was applied between runs. Proteins with |log_2_ fold change| > 0.58 and *q* < 0.05 (calculated using Student’s *t*-test as implemented in Spectronaut) were considered as differentially expressed proteins (DEPs).

## Results and Discussion

### Global Multi-Omics Data

**FIGURE 1:**
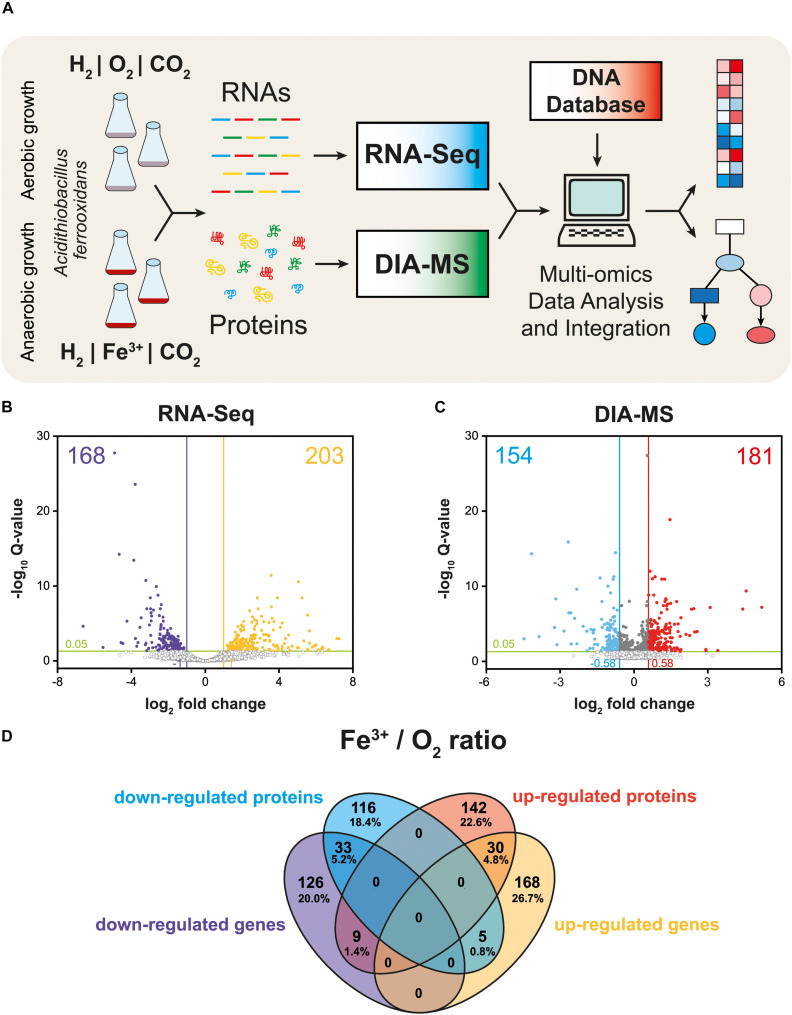
Multi-omics analysis of aerobically and anaerobically grown *At. ferrooxidans* cells with hydrogen as an electron donor. Experimental design including cultivation, next-generation sequencing, quantitative proteomics, and bioinformatic data analysis **(A)**. Volcano plot representing all expressed genes **(B)**. Volcano plot representing all identified proteins **(C)**. Closed circles indicate gene transcripts and proteins that changed significantly (*q* < 0.05); colored circles indicate significant fold change (|log_2_ fc| > 1 for gene transcripts, and 0.58 for proteins, respectively). Venn diagram displays significant differentially expressed genes (DEGs) and proteins (DEPs) values (*q* < 0.05) with |log_2_ fc| > 1 for gene transcripts, and 0.58 for proteins, respectively **(D)**.

**FIGURE 2:**
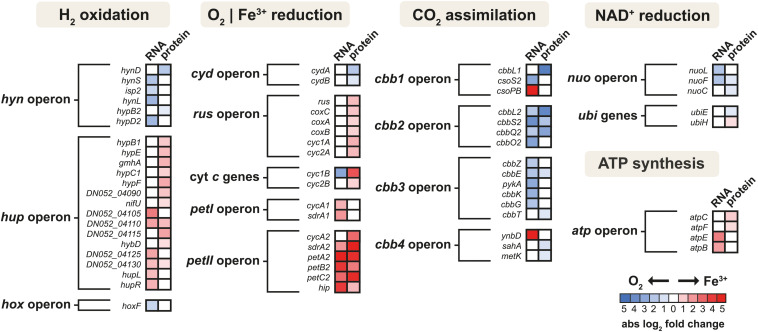
The heat map of products of energy metabolism-related gene clusters that were differentially expressed in aerobically and anaerobically grown *At. ferrooxidans* cells with hydrogen as an electron donor. Shown are only genes and proteins that changed significantly (*q* < 0.05) with absolute log_2_ fold change > 1 for gene transcripts and 0.58 for proteins.

**FIGURE 3:**
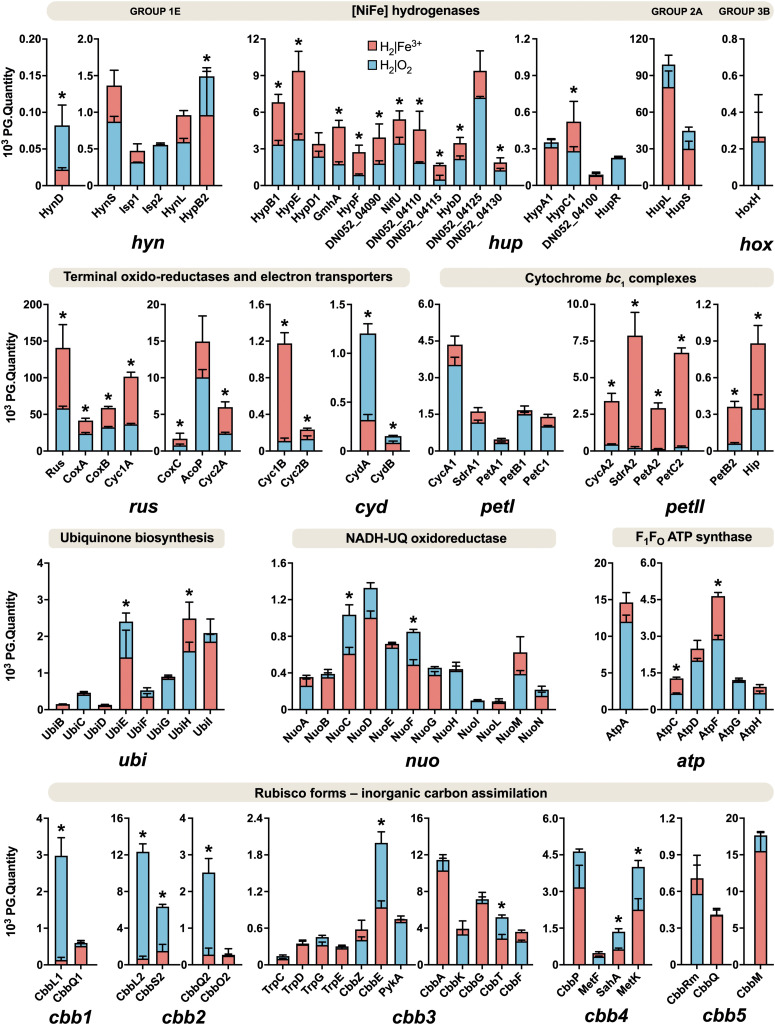
Relative abundances of energy metabolism proteins in aerobically and anaerobically grown *At. ferrooxidans* cells with hydrogen as an electron donor. Blue bars represent aerobic growth (electron acceptor: oxygen), and red bars represent anaerobic growth (electron acceptor: ferric iron). An asterisk indicates a significant change between the aerobic and anaerobic growth (|log_2_ fold change| > 0.58 and *q* < 0.05). Error bars are standard deviations of triplicate analyses.

**FIGURE 4:**
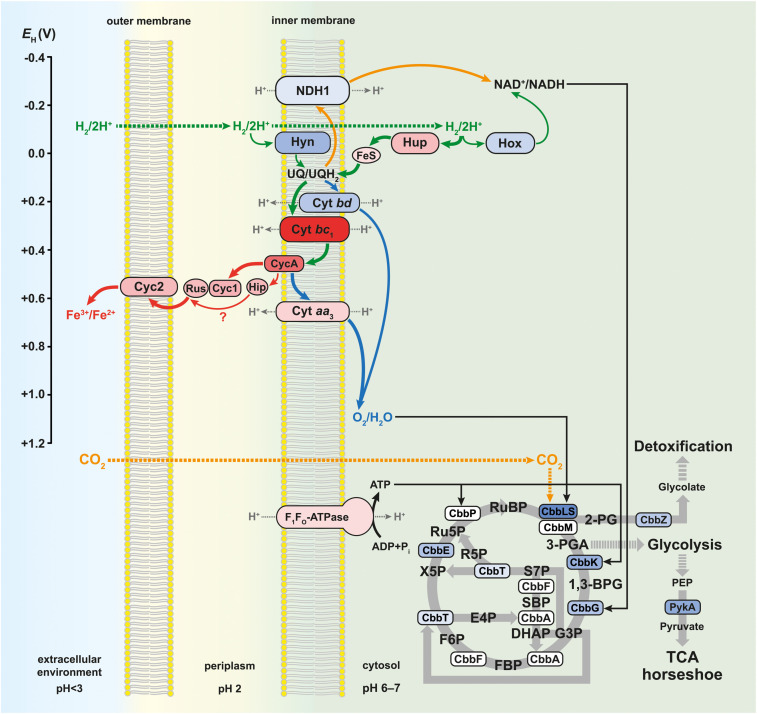
Model of aerobic and anaerobic metabolism of hydrogen connected with carbon dioxide assimilation in *At. ferrooxidans*. Solid green arrows indicate direct electron transfer, solid orange arrows indicate reverse electron transfer, and solid gray arrows indicate enzymatic reactions during the aerobic and anaerobic metabolism. Solid blue arrows indicate direct electron transfer during aerobic metabolism, while solid red arrows indicate direct electron transfer during anaerobic metabolism. The thickness of the colored arrows corresponds to the pathway significance according to the relative protein abundance in Figure 3. Dotted gray arrows indicate proton transfer, dotted green arrows indicate hydrogen influx, and dotted orange arrows indicate carbon dioxide influx. The color of proteins and multiprotein complexes corresponds to the log_2_ fold changes in Figure 2. The redox potential values (*E*_H_) for individual proteins and multiprotein complexes are referenced in the text. 2-PG, 2-phosphoglycolate; 3-PGA, 3-phosphoglycerate; 1,3-BPG, 1,3-bisphosphoglycerate; G3P, glyceraldehyde 3-phosphate; DHAP, dihydroxyacetone phosphate; SBP, sedoheptulose 1,7-bisphosphate; S7P, sedoheptulose 7-phosphate; E4P, erythrose 4-phosphate; FBP, fructose 1,6-bisphosphate; F6P, fructose 6-phosphate; R5P, ribose 5-phosphate; X5P, xylulose 5-phosphate; Ru5P, ribulose 5-phosphate; RuBP, ribulose 1,5-bisphosphate; PEP, 2-phosphoenolpyruvate; TCA, tricarboxylic acid.

The schematic of the multi-omics approach used is shown in Figure 1A. This identified a total of 3,169 gene transcripts (98.4% coverage; a total of 3,219 coding sequences in *At. ferrooxidans* CCM 4253 genome) and 8,949 proteotypic (non-shared) peptides (Supplementary Table 1) representing 1,427 proteins (Supplementary Table 2), of which 1,412 proteins (46.2% coverage; a total of 3,059 protein-coding genes in *At. ferrooxidans* CCM 4253 genome) were quantified based on at least one proteotypic peptide in at least 50% measurements across the study. By comparing the anaerobic versus aerobic H_2_-oxidizing *At. ferrooxidans* cultures, a total of 371 DEGs (Supplementary Table 3) and 335 DEPs (Supplementary Table 4) were found. Of these, 203 DEGs and 181 DEPs were up-regulated during anaerobic growth on H_2_, while 168 DEGs and 154 DEPs were down-regulated (Figures 1B,C). Furthermore, 168 DEGs and 142 DEPs were uniquely up-regulated, and only 30 DEGs/DEPs showed mutual upregulation, while 126 DEGs and 116 DEPs were uniquely down-regulated and only 33 DEGs/DEPs exhibited mutual downregulation. Thus, the correlation between the DEGs and DEPs identified by each omics method was modest; 4.8% upregulation and 5.2% downregulation (Figure 1D), respectively. This relatively low overlap between mutually regulated genes and their products under the same growth conditions can be attributed to various factors, such as different half-lives and post transcription machinery (Haider and Pal, 2013). The most abundant proteins, representing more than 1% of the total protein in *At. ferrooxidans*, were the same for aerobic and anaerobic growth with H_2_. Among the most represented proteins were those encoded by the *hup* operon (small and large subunits of uptake [NiFe] group 2a hydrogenase involved in H_2_ oxidation), and the *rus* operon (cytochrome *c*_552_ and rusticyanin involved in electron transport during iron oxido-reduction, and two subunits of terminal *aa*_3_ oxidase involved in O_2_ reduction). Furthermore, outer membrane proteins (OmpA and Omp40, which increase cell hydrophobicity and help adhesion), and the GroEL/ES chaperonin system that functions as a protein folding cage, accounted for > 1% of total proteins (Supplementary Figure 1). The electron acceptor-dependent changes in the expression of gene cluster products (RNAs and proteins) that are involved in the energy metabolism of *At. ferrooxidans* growing on H_2_ are shown in Figure 2. The relative abundances of the energy metabolism proteins under both growth conditions are shown in Figure 3. Based on results obtained in this work, a model of aerobic and anaerobic metabolism of H_2_ connected with CO_2_ assimilation in the extremophile *At. ferrooxidans* is proposed (Figure 4).

### Molecular Hydrogen Metabolism

Aerobic and anaerobic oxidation by acidophilic bacteria such as *At. ferrooxidans* contributes to the global H_2_ cycle and may promote microbial productivity in oligotrophic environments. The redox potentials (*E*_H_) of the H_2_/2H^+^ and O_2_/H_2_O couples are −118 mV (calculated using the Nernst equation) and +1,120 mV at pH 2 (Bird et al., 2011), respectively. To avoid ferric iron insolubility (at pH > 3), the iron oxidation and reduction occur on the outer membrane or periplasm of *Acidithiobacillus* spp., which have a similar pH to that of the acidic bathing liquors in which these obligate acidophiles thrive. The *E*_H_ value of the Fe^2+^/Fe^3+^ couple in acidic, sulfate-rich solutions at pH 2.0 was measured to be +663 mV (Johnson et al., 2017). The net potential differences between H_2_ as electron donor and O_2_ or Fe^3+^ as electron acceptors are ∼1,238 and ∼781 mV, respectively. While the *E*_H_ value of the Fe^2+^/Fe^3+^ varies with pH and whether one (or both) species of iron are complexed, it is always less electro-positive than the O_2_/H_2_O couple, inferring that anaerobic oxidation of H_2_ coupled to Fe^3+^ yields marginally less energy than oxidation coupled to O_2_. In addition, during aerobic growth, pH values remained steady (around 1.8), while slightly decreasing from 2.0 to 1.8 during anaerobic growth, which supported the theoretical equations and the formation of water under aerobic and protons and ferrous iron under anaerobic conditions, respectively. We have found all groups (1–4) of [NiFe] hydrogenases in the *At. ferrooxidans* CCM 4253 genome sequence, identical to those from the type strain (Valdés et al., 2008). However, our proteomic analysis revealed only the presence of three [NiFe] hydrogenases in *At. ferrooxidans*, namely group 1 Hyn, group 2 Hup, and group 3 Hox (Figure 3). The small subunit of Hyn (HynS) has a twin-arginine transport (TAT) signal sequence [ST]-R-R-x-F-L-K, it is transported across the membrane by the TAT system and is anchored to the membrane on the periplasmic side. The Hup and Hox have no signal for transport across the membrane, which makes them likely cytoplasmic enzymes. A comparison of protein abundance showed that H_2_ oxidation is predominantly ensured by the cytoplasmic Hup (Figure 3). This finding is consistent with earlier observations where 90% of the total hydrogenase activity was recovered from soluble fraction, while 12% of the total activity was found in the membrane fraction of *At. ferrooxidans* (Fischer et al., 1996). The *hynS-isp1-isp2-hynL* structural genes encode the inner-membrane respiratory H_2_-uptake [NiFe] group 1e hydrogenase (Isp-type), which is typically present in phototrophic and chemotrophic bacteria capable of respiratory sulfur oxidation and reduction. According to HydDB database, the group 1e-hydrogenase is bidirectional, O_2_-sensitive (some with a tolerance to microoxic conditions) enzyme thought to be involved in hydrogenotrophic respiration using sulfur as terminal electron acceptor (Søndergaard et al., 2016). The cluster also includes maturation-related genes, which are essential for respiratory hydrogenase complex formation. The structural genes *hynS*, *isp2*, *hynL*, and the maturation-related gene *hypD2*, were all significantly induced during aerobic growth with H_2_. At the protein level, only maturation related HynD and HynB2 were increased during aerobic H_2_ oxidation (Figure 2). Given the overall low abundance (Figure 3), it would be anticipated that the membrane-bound respiratory Hyn only complements H_2_ oxidation coupled to oxygen or ferric iron reduction. Transport of electrons from [NiFe] group 1–2 hydrogenases into the respiratory chain to quinones is assumed to be mediated by a protein carrying the [FeS] center (Islam et al., 2019). Therefore, the electrons derived from H_2_ oxidation in the active [NiFe] center of an enzyme are further transported up to the Ips2 subunit, [FeS]-binding protein (4Fe-4S ferredoxin-type), which transfers them to quinones in the cytoplasmic membrane (Figure 4). On the other hand, the main H_2_ oxidation pathway seems to be mediated by a soluble uptake Hup which was previously purified from *At. ferrooxidans* ATCC 19859 (Fischer et al., 1996). The enzyme consisting of two subunits (large of 64 kDa and small of 34 kDa) corresponds to alternative and sensory H_2_-uptake [NiFe] group 2a hydrogenase (*Cyanobacteria*-type), which is widespread among *Cyanobacteria* and aerobic soil bacteria. The group 2a hydrogenase is membrane-associated, unidirectional (some with high-affinity), O_2_-tolerant enzyme suggested to be involved in hydrogenotrophic respiration using O_2_ as the terminal electron acceptor (Søndergaard et al., 2016). The Hup reacted with methylene blue and other artificial electron acceptors, but not with NAD^+^, and has optimum activity at pH 9 and 49°C (Fischer et al., 1996). This cyanobacterial-like hydrogenase showed the characteristics of uptake [NiFe] hydrogenases as determined by EPR and FTIR (Schröder et al., 2007). The *hupL-hupS* structural genes encode the uptake [NiFe] group 2a hydrogenase. Similar to group 1e, the cluster contains maturation-related genes, in addition to transcriptional factor *hupR* and genes with unknown function. Hup showed about a 100-fold higher relative protein abundance compared to other hydrogenases in *At. ferrooxidans* growing on H_2_ under both growth conditions (Figure 3). Also, both subunits of Hup were among the most abundant proteins. The small subunit represented 2.5% (aerobic growth) and 1.4% (anaerobic growth) of total protein, while the large subunit represented 5.6% (aerobic growth) and 3.6% (anaerobic growth) of total protein (Supplementary Figure 1). In addition, gene loci with unknown function DN052_04105–04110 and DN052_04125–04130 were induced during anaerobic growth, as well as genes encoding the structural subunit and transcriptional regulator, *hupL* and *hupR*, respectively (Figure 2). Furthermore, the elevated levels of maturation proteins (HypB1, HypE, GmhA, HypC1, HypF, HybD), proteins with unknown functions (DN052_04090, DN052_04110, DN052_04115), iron-sulfur proteins (NifU and DN052_04130) were detected during anaerobic growth (Figure 2). There are two potential candidates of protein carrying the [FeS] center in the *hup* operon. The first candidate is the *nifU* gene, a locus DN052_04095, which encodes the Rieske protein with [2Fe-2S] iron-sulfur domain. Rieske proteins are components of cytochrome *bc*_1_ (proteobacteria) and *b*_6_*f* (cyanobacteria) complexes that are responsible for electron transfer in the respiratory chain (ten Brink et al., 2013). Another candidate is near the *hupLS* genes encoding the structural subunits, a locus DN052_04130, which encodes putative high potential iron-sulfur protein (HiPIP). HiPIPs are a specific class of high-redox potential [4Fe-4S] ferredoxins that are commonly found in various bacteria as periplasmic electron carriers between the *bc*_1_ complex and the reaction center or a terminal oxidase (Nouailler et al., 2006). One or both of these proteins are likely the missing link in the electron transfer between the H_2_ oxidation in the cytoplasm and the respiratory chain represented by the ubiquinone/ubiquinol (UQ/UQH_2_) pool in the cytoplasmic membrane. The ubiquinol molecule produced by the hydrogenases of the respiratory chain can diffuse within the membrane bilayer to the cytochrome ubiquinol oxidase or the cytochrome *bc*_1_ complex (Figure 4). The expression of the *hoxF* gene encoding the alpha subunit of cofactor-coupled bidirectional [NiFe] group 3b hydrogenase (NADP-coupled) was significantly increased during aerobic growth with H_2_ (Figure 2). The alpha subunit possesses a [4Fe-4S] ferredoxin domain that provides H_2_/H^+^ production. The group 3b hydrogenase is a cytosolic, bidirectional, O_2_-tolerant enzyme encoded in many diverse bacterial and archaeal phyla that was proposed to directly couple oxidation of NADPH to fermentative generation of H_2_. The reverse reaction may also occur. Some enzymes have been controversially proposed to harbor sulfhydrogenase activity (Søndergaard et al., 2016). At the protein level, however, only the beta subunit HoxH was detected whose low level was not significantly altered depending on the terminal electron acceptor (Figure 3). Group 3b hydrogenase may minorly oxidize H_2_ to form NAD(P)H, which can be further utilized, e.g., in the Calvin cycle (Figure 4). The reverse role of the group 3b hydrogenase could be NAD(P)^+^ recycling using protons or water and therefore serves as an electron sink under high reduction conditions (Valdés et al., 2008). None of the respiratory H_2_-evolving [NiFe] group 4 hydrogenase subunits were identified during either aerobic or anaerobic growth with H_2_. Non-hydrogenase catalytic subunit sequences were found in the *hyfBCEFGI* cluster (DN052_15040–15065) using an accurate classifier and a curated database of hydrogenases HydDB (Søndergaard et al., 2016). *At. ferrooxidans* thus probably has only three [NiFe] hydrogenases representing the groups 1e, 2a, and 3b. As *At. ferrooxidans* ATCC 21834 was shown to grow on formate when the substrate supply was growth limiting (Pronk et al., 1991), another role for the membrane-bound Hyf complex may involve the oxidation of formate (Valdés et al., 2008).

### Molecular Oxygen Reduction

In this work, a significant increase in both the subunits I and II (CydA and CydB) of cytochrome *bd* ubiquinol oxidase was observed during aerobic growth with H_2_ (Figure 2). The bioenergetic function of cytochrome *bd* is to conserve energy in the form of ΔμH^+^, although the H^+^/e^–^ ratio is one because the cytochrome *bd* does not pump protons. In addition to the generation of PMF, the *bd*-type oxygen reductase gives bacteria some other vital physiological functions. The apparent redox potentials of *b*_558_, *b*_595_, and *d* for cytochrome *bd* oxidase for *E. coli* at pH 7 were shown to be in the range of +176, +168, +258 mV, respectively. Furthermore, it was reported that these values are sensitive to pH, so they increase with decreasing pH (Borisov et al., 2011). None of the cytochrome *bo*_3_ ubiquinol oxidase subunits were identified during either aerobic or anaerobic growth with H_2_. On the other hand, the CoxC, CoxA, and CoxB subunits of *aa*_3_ cytochrome *c* oxidase were significantly increased during anaerobic growth with H_2_ (Figure 2). The *a*-type cytochromes in *At. ferrooxidans* also have pH-dependent redox potential of +725 mV and +610 mV at pH 3.2, and +500 mV and +420 mV at pH 7 (Ingledew and Cobley, 1980). The higher abundance of cytochrome *aa*_3_ oxidase in O_2_-free conditions was likely related to co-expression with other genes within the *rus* operon (Figure 2). Moreover, cytochrome *aa*_3_ may serve as a residual O_2_ scavenger to inhibit the degradation of O_2_-sensitive proteins and thus support anaerobic growth. The cytochrome *aa*_3_ subunits I and II represented the most abundant proteins under both aerobic (1.4 and 1.9% of total proteins, respectively) and anaerobic conditions (1.9 and 2.7%) in which their reductase function could be utilized (Supplementary Figure 1). By comparing the quantity of both terminal oxidases, the proportion of the *aa*_3_-type is much higher than that of the *bd*-type (Figure 3). We hypothesize that *At. ferrooxidans* reduces O_2_ to H_2_O in two parallel pathways during aerobic H_2_ oxidation. The first O_2_ reduction pathway includes cytochrome *bd* ubiquinol oxidase, which acquires electrons directly from the UQ/UQH_2_ pool. However, there is only one PMF-generating complex in this pathway, so less energy is conserved in the form of ATP and NADH. The second O_2_ reduction pathway includes cytochrome *aa*_3_ oxidase, which acquires electrons from the UQ/UQH_2_ pool via cytochrome *bc*_1_ complex by cytochrome *c*_4_. There are already two PMF-generating complexes in this pathway and therefore provide more energy (Figure 4). *At. ferrooxidans* genome sequence contains two operons encoding the cytochrome *bc*_1_ complex (*petI–II*). Products of the *petI* operon are known to be important in reverse electron flow to NADH-UQ oxidoreductase (NDH1) in aerobic Fe^2+^ oxidation, and *petII* products are likely to complement electron transfer from UQ/UQH_2_ pool to terminal oxidase in aerobic RISCs oxidation (Quatrini et al., 2009). By comparing the quantity of both cytochrome *bc*_1_ complexes during aerobic growth with H_2_, the proportion of the *bc*_1_ complex I is higher than that of the *bc*_1_ complex II, which is almost undetectable (Figure 2). Thus, it is likely that electrons from the UQ/UQH_2_ pool are transported to the terminal *aa*_3_-type oxidase via *bc*_1_ complex I (PetA1B1C1) by membrane-associated cytochrome *c*_4_ (CycA1). It would mean that the *bc*_1_ complex I can transfer electrons even in a direct flow to the terminal oxidase following the redox potential gradient, and not in reverse flow to the NDH1 complex, when the electron donor is H_2_.

### Ferric Iron Reduction

In this work, almost all the *rus* operon products were significantly elevated at the protein level in *At. ferrooxidans* during anaerobic growth with H_2_ coupled to Fe^3+^ reduction (Figure 2). Significant changes were observed in the synthesis of rusticyanin, Cyc1A, and Cyc2A. Rusticyanin and Cyc1A represented the most abundant proteins in aerobically (3.3 and 2.1% of total proteins, respectively) and anaerobically (6.4 and 4.6%) grown *At. ferrooxidans* cells (Supplementary Figure 1). High level of soluble acid-stable 28 kDa *c*-type cytochrome was observed in *At. ferriphilus* JCM 7811 grown anaerobically on H_2_ coupled to Fe^3+^ reduction. Also, the presence of iso-rusticyanin in cells of this related bacterium grown anaerobically on H_2_ was detected by immunostaining (Ohmura et al., 2002). The *At. ferrooxidans* genome sequence contains a two-gene cluster DN052_01245–DN052_01250 encoding *c*-type cytochromes that are homologs of the Cyc1A and Cyc2A. The new *c*-type cytochromes (Cyc1B and Cyc2B) have been discovered in *At. ferrooxidans*^T^ during anaerobic growth with S^0^ coupled to Fe^3+^ reduction. Their relative protein abundances under anaerobic conditions were even higher than the levels of their homologs encoded by the *rus* operon (Norris et al., 2018). In this work, both Cyc1B and Cyc2B were significantly increased during anaerobic growth of *At. ferrooxidans* with H_2_ coupled to Fe^3+^ reduction (Figure 2). However, their relative abundances were considerably lower compared to Cyc2A and especially Cyc1A (Figure 3). The significance of the role of these homologs in respiratory Fe^3+^ reduction may be related to specific strains, substrate, or longer adaptation, though cytochromes Cyc2 and Cyc1 seem to play an essential role in the electron transport and mechanism of Fe^3+^ reduction. The *E*_H_ of *c*-type cytochromes from *At. ferrooxidans* typically are +560 mV Cyc2 (pH 4.8), +385 and +485 mV Cyc1 (pH 3), +510 and +430 mV CycA (pH 4), and +680 mV for blue-copper protein rusticyanin (pH 3.2) (Bird et al., 2011). Moreover, the formation of a complex between rusticyanin and Cyc1 decreases the rusticyanin redox potential by more than 100 mV, which facilitates electron transfer (Roger et al., 2012). Thus, the electrons needed for Fe^3+^ reduction are delivered via a cascade of periplasmic and membrane-associated electron carriers. Periplasmic rusticyanin forming a complex with Cyc1 receives electrons from the inner membrane-anchored cytochrome CycA and transmits them to outer membrane cytochrome Cyc2. Extracellular Fe^3+^ is then reduced from the outside of the outer membrane by Cyc2 (Figure 4). All homologs Cyc1 and Cyc2 are considered, i.e., variants A and B. Cytochrome *c*_4_ (CycA) accepts electrons from the inner-membrane cytochrome *bc*_1_ complex, which transfers them from the UQ/UQH_2_ pool. During anaerobic growth with H_2_, the complete *petII* operon was strongly induced at the level of transcription and protein synthesis (Figure 2). On the other hand, a significant increase in the expression of two genes of the *petI* operon was also detected. By comparing the protein quantity of both cytochrome *bc*_1_ complexes during anaerobic growth on H_2_, the proportion of the *bc*_1_ complex II is much higher than that of the *bc*_1_ complex I (Figure 3). The electrons appear to be transported from the UQ/UQH_2_ pool to the *bc*_l_ complex II (PetA2B2C2), which further transfers them to CycA2 (Figure 4). Nevertheless, a slight involvement of the *bc*_1_ complex I (PetA1B1C1) and CycA1 in electron transport might be expected, as they are also present in *At. ferrooxidans* during anaerobic H_2_ oxidation (Figure 3), which is consistent with previous results in the anaerobic S^0^ oxidation (Kucera et al., 2016a). The involvement of high potential iron-sulfur protein (Hip, formerly Iro) in electron transport remains an issue. The Hip is part of the *petII* operon and was significantly increased during anaerobic H_2_ oxidation coupled to Fe^3+^ reduction (Figure 2), and also during anaerobic S^0^ oxidation coupled to Fe^3+^ reduction (Kucera et al., 2016a). The functional form of Hip contains a redox-active [4Fe-4S] cluster, which is usually sensitive to O_2_. This feature may predetermine its function in an anaerobic respiration process. The redox potential of Hip decreases linearly depending on pH in the range 3.5–5, but remains constant at lower and higher pH, and is +550 mV at pH 2 (Bruscella et al., 2005). The same pH dependence of redox potential was observed for rusticyanin (Haladjian et al., 1993) and Cyc1 (Haladjian et al., 1994). From their properties, it is possible that Hip and rusticyanin/Cyc1 function interchangeably to reduce terminal Fe^3+^ reductase in this bacterium (Figure 4). However, the role of Hip in the mechanism of anaerobic H_2_ oxidation in *At. ferrooxidans* may not be essential, due to the relatively non-specific electron transfer and comparison of its quantity to rusticyanin and other *c*-type cytochromes (Figure 3).

### Energy Conservation

Because the *E*_H_ values of UQ/UQH_2_ and NAD^+^/NADH are +110 and –320 mV, respectively, at cytoplasmic pH values (pH 6–7), some electrons coming from substrate oxidation are pushed uphill against the unfavorable redox potential. This reverse electron flow is driven by the PMF (Bird et al., 2011). Three subunits NuoL, NuoF, and NuoC of the NDH1 were induced when *At. ferrooxidans* grew aerobically with H_2_ (Figure 2). To date, there is no evidence that the same mechanism of reverse flow for NADH regeneration is the case for both aerobic and anaerobic respiration. On the other hand, many autotrophic bacteria use multiple electron donors and acceptors, suggesting the existence of a universal pathway for NADH regeneration through uphill transfer connecting to each respiratory chain (Ohmura et al., 2002). Our data support this hypothesis, with the finding of the same level of induction of the majority (10 of 14) of NDH1 subunits when *At. ferrooxidans* used H_2_ as an electron donor and either O_2_ or Fe^3+^ as electron acceptor (Figure 3). On the other hand, the upregulation of a few genes encoding the NDH1 complex under aerobic growth was likely related to the upregulation of other genes of the *cbb* operons involved in CO_2_ assimilation (discussed below), which require reducing equivalents such as NADH. The higher energy gain when the terminal acceptor is O_2_ may lead to an increased reduction of NAD^+^ to NADH, which allows a higher rate of CO_2_ assimilation resulting in higher biomass during aerobic growth. Also, the ubiquinone/menaquinone biosynthesis C-methyltransferase (UbiE) was more abundant under aerobic conditions, whereas 2-octaprenyl-6-methoxyphenyl hydroxylase (UbiH) was more abundant under anaerobic conditions (Figure 2), both of which are involved in the ubiquinone biosynthesis. Although UbiE and UbiH altered in protein quantity depending on electron acceptor, the majority of proteins in this pathway were constitutively synthesized (Figure 3) to provide the required ubiquinone pool for both aerobic and anaerobic respiration (Figure 4). *At. ferrooxidans* conserves energy by producing ATP from ADP via F_1_F_O_ ATP synthase in the presence of a proton gradient (Ingledew, 1982). In this study, significant abundances in epsilon subunit (AtpC) of the F_1_ portion and subunit b (AtpF) of the F_O_ portion were found during anaerobic growth of *At. ferrooxidans* on H_2_ (Figure 3). In addition, genes *atpE* and *atpB* encoding subunits a and c of F_O_ portion, respectively, were increased at their transcript levels (Figure 2).

### Carbon Metabolism

Chemoautotrophic acidophilic bacteria use different pathways for inorganic carbon (Ci) assimilation to produce complex organic compounds. Five operons (*cbb1-5*) in the *At. ferrooxidans* genome, encoding enzymes and structural proteins involved in Ci assimilation via the Calvin-Benson-Bassham (CBB) pathway, have been described (Esparza et al., 2010, 2019). In this work, we investigated changes in the expression of all five *cbb* operons during aerobic and anaerobic growth of *At. ferrooxidans* with H_2_ (Figure 2). Interestingly, although the concentration of CO_2_ was greater under anaerobic conditions, we detected an upregulation of *cbb* genes under aerobic conditions, which may indicate not only their CO_2_-depending regulation, but also an impact of other factors, such as O_2_ concentration. A key enzyme in the CBB pathway is cytoplasmic ribulose bisphosphate carboxylase/oxygenase (Rubisco). Of all Rubisco forms, a form II (CbbM) was the most abundant under both growth conditions, although its abundance did not change significantly (Figure 3). The form II seems to be synthesized constitutively, independent of O_2_ concentration. Rubisco form II has a weak affinity for CO_2_ (Esparza et al., 2019), which might explain its higher abundance compared to IAc and IAq. Nevertheless, a large subunit of the form IAc (CbbL1) and both subunits of the form IAq (CbbL2S2) were significantly more abundant during aerobic growth of *At. ferrooxidans* with H_2_ (Figure 2). Both Rubisco IAc and IAq forms appear to be O_2_ dependent. As carboxylation and oxidation of RuBP coincide, both reactions may compete in the same active place. Enzymes passing the carbon from 3-phosphoglycerate produced by Rubisco via the CBB and glycolysis pathways to pyruvate and glycogen metabolism pathways are encoded by the *cbb3* operon (Esparza et al., 2019), the expression profile of which is shown in Figure 2. The initial Ci assimilation pathways during H_2_ oxidation are proposed in Figure 4.

## Conclusion

We provide the first overall insight into the mechanisms employed by acidithiobacilli to metabolize hydrogen in low-pH aerobic and anaerobic environments. The model presented here describes the molecular hydrogen metabolism and the energy conservation associated with the assimilation of inorganic carbon. This study is a fundamental step in identifying elements of metabolic pathways when *At. ferrooxidans* utilizes hydrogen as an electron donor and may further be a starting point for characterizing the physiology of hydrogen metabolism and ferric iron reduction in other mesophilic acidophiles.

## Data Availability Statement

The datasets presented in this study can be found in online repositories. The transcriptomic data are available in Gene Expression Omnibus (GEO) repository under reference number GSE154815. The mass spectrometry proteomics data are available via ProteomeXchange with identifier PXD020361.

## Author Contributions

JK and DBJ designed the study. JK and EP conducted the laboratory experiments. JL performed RNA sequencing and data analysis. KM performed the LC-MS/MS analysis. PB performed the analysis of MS/MS data. JK, MM, and OJ were involved in data analysis and biological interpretation of the results. JK prepared the manuscript. SH, EP, and DBJ edited the manuscript. All authors contributed to the article and approved the submitted version.

## Conflict of Interest

The authors declare that the research was conducted in the absence of any commercial or financial relationships that could be construed as a potential conflict of interest.

## Footnotes

1 www.maxquant.org

## Supplementary Material

Supplementary Figure 1 Proteins representing more than 1% of the total protein in hydrogen-oxidizing *Acidithiobacillus ferrooxidans* cells. Blue bars represent aerobic growth (electron acceptor: oxygen), and red bars represent anaerobic growth (electron acceptor: ferric iron). Labels show percentages of total protein in each growth condition. Error bars are standard deviations of triplicate analyses.

Supplementary Table 1 List of all quantified peptides in DIA-MS proteomics analysis.

Supplementary Table 2 List of all quantified proteins in DIA-MS proteomics analysis.

Supplementary Table 3 Differentially expressed genes (DEGs) in aerobically and anaerobically grown *At. ferrooxidans* cells with hydrogen as an electron donor.

Supplementary Table 4 Differentially expressed proteins (DEPs) in aerobically and anaerobically grown *At. ferrooxidans* cells with hydrogen as an electron donor.
