# Supplementary figures and images for: A Model of Aerobic and Anaerobic Metabolism of Hydrogen in the Extremophile Acidithiobacillus ferrooxidans
Source: Front Microbiol. 2020 Nov 30;11:610836. doi: 10.3389/fmicb.2020.610836 (PMC7735108; doi:10.3389/fmicb.2020.610836)

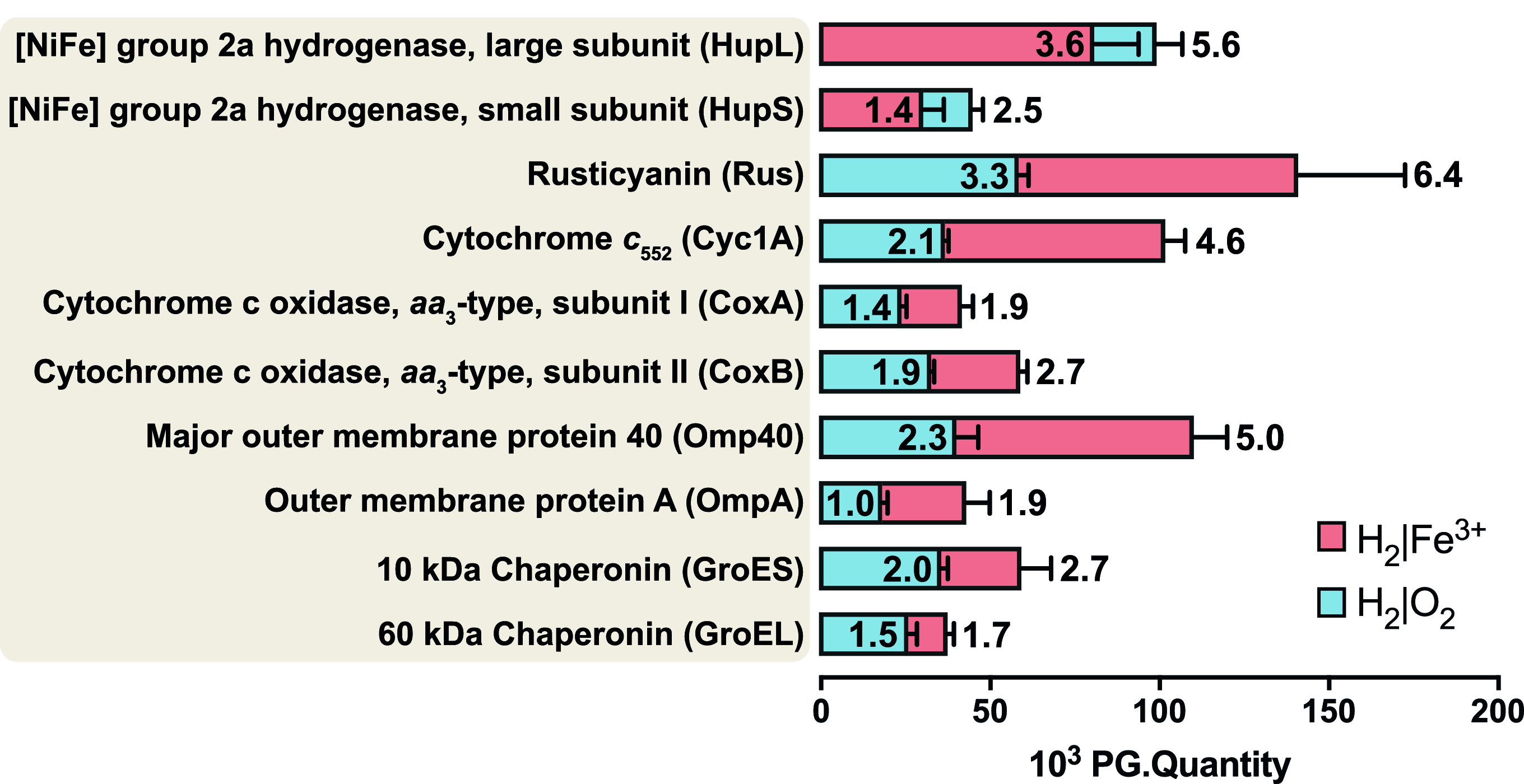

Supplement: Supplementary Figure 1 — Proteins representing more than 1% of the total protein in hydrogen-oxidizing Acidithiobacillus ferrooxidans cells. Blue bars represent aerobic growth (electron acceptor: oxygen), and red bars represent anaerobic growth (electron acceptor: ferric iron). Labels show percentages of total protein in each growth condition. Error bars are standard deviations of triplicate analyses. [file Image_1.TIF]
